# The Edge Factor in Early Word Segmentation: Utterance-Level Prosody Enables Word Form Extraction by 6-Month-Olds

**DOI:** 10.1371/journal.pone.0083546

**Published:** 2014-01-08

**Authors:** Elizabeth K. Johnson, Amanda Seidl, Michael D. Tyler

**Affiliations:** 1 Department of Psychology, University of Toronto, Toronto, Ontario, Canada; 2 Department of Speech, Language, and Hearing Sciences, Purdue University, West Lafayette, Indiana, United States of America; 3 MARCS Institute and School of Social Sciences and Psychology, University of Western Sydney, Sydney, New South Wales, Australia; Massachusetts Institute of Technology, United States of Ameirca

## Abstract

Past research has shown that English learners begin segmenting words from speech by 7.5 months of age. However, more recent research has begun to show that, in some situations, infants may exhibit rudimentary segmentation capabilities at an earlier age. Here, we report on four perceptual experiments and a corpus analysis further investigating the initial emergence of segmentation capabilities. In Experiments 1 and 2, 6-month-olds were familiarized with passages containing target words located either utterance medially or at utterance edges. Only those infants familiarized with passages containing target words aligned with utterance edges exhibited evidence of segmentation. In Experiments 3 and 4, 6-month-olds recognized familiarized words when they were presented in a new acoustically distinct voice (male rather than female), but not when they were presented in a phonologically altered manner (missing the initial segment). Finally, we report corpus analyses examining how often different word types occur at utterance boundaries in different registers. Our findings suggest that edge-aligned words likely play a key role in infants’ early segmentation attempts, and also converge with recent reports suggesting that 6-month-olds’ have already started building a rudimentary lexicon.

## Introduction

Human speech is characterized by a lack of fully reliable cues to word boundaries, since the gestures made to articulate one speech sound typically blend seamlessly into the gestures made to produce neighboring speech sounds, even when adjacent speech sounds span a word boundary (e.g., [1]). For this reason, when we listen to an unfamiliar language, word boundaries are virtually impossible to identify unless they are aligned with a major prosodic boundary, such as those that typically occur at the end of a clause or sentence (e.g., [2]). Adults readily perceive utterance-medial word boundaries in their native language in large part because they have experience-based expectations concerning how words typically sound (e.g., [3], [4], [5]). But when and how do children initially begin to perceive word boundaries or begin to “segment” words from speech? In the current study, we examine early word segmentation by considering the role that infants’ attention to major prosodic boundaries might play in their initial segmentation attempts.

One of the first studies to examine the emergence of word segmentation abilities in infancy was carried out by Jusczyk and Aslin [6]. In a familiarization phase, 7.5-month-old English learners heard isolated repetitions of a word when they oriented towards a side light mounted above a loudspeaker. Two words (e.g., ‘dog’ and ‘cup’) were presented on alternating trials and familiarization ended when infants had accrued 30 seconds of looking time to each of the two words. Using the same procedure, their looking times were tallied for passages that either did or did not contain the familiarized words. Infants preferred to listen to passages containing the familiarized words versus passages not containing the familiarized words, indicating that they could recognize the familiarized words in fluent speech. In another experiment, infants were first familiarized with fluent passages containing two target words and then tested on their ability to recognize those words produced in isolation. Once again, 7.5-month-olds succeeded in recognizing the familiarized word forms. Importantly for the purposes of the current study, *6-month-olds* tested using the exact same procedure showed no evidence of recognizing the familiarized words. This led Jusczyk and Aslin to conclude that the ability to segment words from speech emerges some time between 6 and 7.5 months of age.

The emergence of word segmentation skills in early infancy is now firmly established, as numerous studies have found results compatible with Jusczyk and Aslin (e.g., [7], [8], [9], [10], [11]). However, the question of precisely how infants first develop the ability to segment words from speech is still under investigation. Research suggests that, from a very young age, infants begin to use the same sort of strategies that adult listeners use to segment words from speech. For example, most content words in English begin with a strong syllable [12]. Accordingly, adult English speakers are biased to perceive strong syllables as word onsets [13]. By 7.5 to 9 months of age, English-learning infants are already aware that strong syllables tend to mark word onsets [14], and they use this information to segment words from speech (e.g., [8], [15]). Another language-specific sound structure cue adults rely on is probabilistic phonotactics. Statistically speaking, some sequences of phonemes are far more likely to occur within words than across word boundaries. By 9 months of age, English-learning infants are aware of English phonotactics [16] and use this information to segment words from speech [9]. Thus, it appears that by 7.5 to 11 months of age, infants already rely on the same sort of probabilistic sound structure and statistical cues that adults rely on to segment words from speech.

Although it is clear that young infants begin to approximate adult-like language-specific segmentation strategies very early in life, it is not yet at all clear how they initially acquire these strategies. Perhaps the most intuitive explanation for how infants overcome the word segmentation problem is that they learn how words sound by attending to words in isolation, however, such a solution does not seem plausible given corpus work revealing the relative scarcity of one-word utterances in infant-directed speech (e.g., [17], [18]). Further, even if infant-directed speech did contain a large proportion of one-word utterances, young infants would have no way of distinguishing between, for example, a multi-syllabic utterance containing many short words and a multi-syllabic utterances containing only one long word (see [19], for discussion). For these reasons, theories relying solely on attention to one-word utterances have been, for the most part, abandoned as untenable (see, however, [20]).

Another more viable explanation for how infants begin segmenting words from the input involves tracking transitional probabilities between syllables. Support for this view has been provided by both behavioural studies (e.g., [21], [22]) as well as corpus analyses [23]. According to this view, infants carefully attend to their language input and store information about the likelihood of one syllable following another. Highly probable syllable transitions are likely to belong to the same word, whereas less probable syllable transitions are more likely to belong to different words. The attractiveness of this hypothesis is that it offers a language-general explanation for infants’ first word segmentation attempts. Once a sufficient number of words have been segmented from speech by using this proposed language-general segmentation strategy, infants could begin to work out what sound structure properties typify the words in their language [19], [23], [25], [26]. For example, from the words that English learners initially extract by tracking transitional probabilities between syllables, they could notice that most words begin with a strong syllable or tend to contain certain phoneme sequences. In this way, the use of a language-general bottom-up segmentation strategy could lead to the establishment of experience-based language-specific segmentation strategies. Support for this view is provided by artificial language learning studies showing that 5- to 6-month-old infants can track transitional probabilities between syllables before they have learned the language-specific sound structure cues signalling word boundaries in their native language [19], [24], [27], [28].

At the same time, despite strong evidence in support of a transitional-probability tracking explanation for infants’ initial word segmentation attempts, there are some indications that attention to transitional probabilities between syllables alone may not always be an optimal segmentation strategy for very young infants. First, the computational demands of tracking transitional probabilities between syllables in natural language may make this solution much more difficult than researchers initially supposed (e.g., [29]). Although it is clear that infants are adept at tracking transitional probabilities between syllables when presented with simplified stimuli in the lab, it is not yet clear whether this ability is robust enough to scale up to the complexity of natural language (see [30] for discussion). Second, it may be the case that syllable tracking works well in some but not all languages, since the syllable is not the primitive in all languages (e.g., [4]), and the sonority profiles of syllables vary across languages [31]. Third, some have suggested that infants may be able to learn the typical stress pattern of their native language before they begin segmenting words from speech [32]. And fourth, although Shukla, White, and Aslin [28] have shown that 6-month-olds use TPs to segment words from an artificial language containing convergent prosodic and visual cues to word boundaries, infants just under 6 months of age appear unable to use transitional probabilities alone to segment words from speech. This is the case even when infants are presented with a highly simplified artificial language. For example, 5.5-month-olds appear unable to track transitional probabilities between syllables even in a simplified artificial language containing only four words with uniform syllable structure without some additional cue such as uniformity in word length [27]. For all of these reasons, it is important to consider that infants may employ alternative (or additional) solutions to segment their first words from speech.

Attention to utterance-level prosody could provide language learners with a highly reliable cues to word boundaries in spoken language [2], [10], [33]–[36]. This is because all utterances produced in natural languages exhibit a perceptually salient hierarchically organized prosodic structure. At the highest level of the Prosodic Hierarchy [37] are utterances. Embedded within utterances are smaller prosodic units, including Intonational Phrases, Phonological Phrases, and Prosodic Words (listed in descending order, [37]). In all languages, utterance and phrase boundaries are acoustically well defined and align with word boundaries. Units along the Prosodic Hierarchy may be marked by pauses and/or pitch resets, vowel lengthening, and other acoustic cues, which are larger and more numerous at higher levels in the hierarchy than at lower levels. Given the ample acoustic marking of utterance and phrase boundaries and their alignment with word boundaries, detection of utterance and phrase boundaries could therefore serve as a deterministic cue to word boundaries, providing the listener with a strong language-general strategy for finding word boundaries in speech. Thus, by attending to word boundaries at utterance edges, infants could begin to learn the language-specific probabilistic cues marking utterance- and phrase-internal word boundaries. Note that use of utterance or phrase edge cues to segment words from speech would not necessarily preclude infants’ use of other additional cues to segment words from speech.

The literature contains substantial support for the feasibility of this hypothesis. Very young infants are highly sensitive to prosodic units. Newborn infants are able to discriminate between lists of two-syllable sequences extracted from longer utterances that were produced as disyllabic words versus lists of two syllable sequences that were produced as parts of longer words with an intervening Phonological Phrase boundary [38], [39]. Four-month-olds prefer to listen longer to utterances containing pauses inserted at intonation phrase boundaries rather than intonation phrase medially [40], while 6-month-olds package speech input in accordance with Phonological Phrase boundaries (e.g., [41]). During the latter half of the first year of life, English learners do not consider syllable sequences as potential word candidates if they span a Phonological Phrase boundary [28], [42], [43] or a Prosodic Word boundary [44]. Finally, adult studies suggest that utterance-level prosody remains a powerful segmentation cue in adulthood, as adults automatically rule out parses that would include syllables spanning a Phonological Phrase boundary as part of the same word [45].

In combination, the studies reviewed above suggest that, over the course of early development, young listeners become increasingly sensitive to the prosodic packaging of speech, and may use this information to locate word boundaries in fluent speech. Indeed, it has been shown that even 7.5-month-olds segment words from speech much more readily when those words occur at the beginning or end of an utterance than the middle of an utterance [10], [35]. In general, these findings all fit well with what Seidl and Johnson have termed the Edge Hypothesis [10], which suggests that utterance edges (much like highly common words, such as the infants’ own name, e.g., [46]) may serve as hotspots, or anchors of reliability, for infants’ initial word segmentation attempts. According to this view, words often flanked by utterance boundaries are amongst the first words infants will segment from speech because utterance boundaries provide the only universal and nearly fully reliable cue to word boundaries. Support for this view is provided by observations of child-caregiver interactions. Caregivers not only tend to provide their children with many utterance-boundary flanked words by speaking in short utterances (e.g. [18], [47]), they also have a tendency to highlight words they are trying to teach to their children by positioning them in utterance-final position [17]. Both of these characteristics of infant-directed speech (IDS) would make it optimally suited for the application of an edge-based word segmentation strategy.

Adult studies also support the notion that utterance edges serve as hot spots for learning, as adults learn phonotactic patterns occurring at major prosodic boundaries faster than they learn those occurring in utterance medial position [48]. Studies with older infants have also shown that several months after they begin segmenting words from speech, older infants nearing their first birthday still rely on utterance edges to locate otherwise difficult-to-perceive word boundaries [35]. However, infants’ use of utterance edges to segment words from natural speech in the first six months of life remains relatively unexplored. It is important to test this period of development because English-learning 6-month-olds have not yet learned any of the language-specific cues to word boundaries such as the placement of stress relative to word boundaries or the phonotactic patterns marking probable word onsets and offsets. We reason that if the Edge Hypothesis is correct, and utterance edges play an important role in enabling infants to make their first passes at segmenting their language input into word-sized units, then infants should show evidence of segmenting words from utterance-aligned positions before they show evidence of segmenting words from utterance-medial position.

In this paper, we present four experiments and a corpus analysis designed to examine the role of utterance edges in infants’ early segmentation attempts. Recall that the seminal work by Jusczyk and Aslin [6] suggested that the ability to segment words from fluent natural speech does not emerge in English learners until they reach 7.5 months of age, however, there is a growing body of evidence suggesting that, under optimal conditions, even 6-month-olds are capable of recognizing spoken words ([46]; see also [49]–[51]). For example, numerous studies have shown that 5- and 6-month-olds can track transitional probabilities between syllables in a simplified artificial language, as long as some sort of convergent rhythmic or prosodic cue is also provided [20], [24], [27], [28]. What is impressive about all of these studies is not that they demonstrate the emergence of early word segmentation and rudimentary word comprehension a month and a half earlier than the classic work by Jusczyk and Aslin [6], but that they show segmentation at an age when English-learning infants have not yet acquired many of the language-specific sound structure cues adults are thought to use to segment words from speech. Thus, one key question regards whether the prevalence of utterance-boundary flanked words in the child’s linguistic input has any role in young infants’ initial segmentation successes.

As mentioned above, we know English-learning 6-month-olds can segment words from natural speech if the target words are preceded by the child’s own name [46]. We also know that 6-month-olds can use a combination of transitional probability cues, visual cues, and prosodic phrasing to segment words from a simplified artificial language [28]. But it is not clear how often names occur in multiword infant-directed utterances and how important a role this information might play in early segmentation attempts. Moreover, it is also not clear whether 6-month-olds are equally adept at segmenting words from natural language as they are at segmenting words from a highly simplified artificial language. For these reasons, the four experiments reported in this paper will take an in-depth look at English-learning 6-month-olds’ ability to segment words from natural speech containing no known proper names. Our goal is not to examine whether infants rely more heavily on TP cues or utterance boundaries to segment words from speech, rather, we simply aim to demonstrate the importance of prosodic edges in infants’ initial segmentation attempts. Thus, all target words in the four experiments we report will be flanked by equally strong transitional-probability cues to word boundaries, however, we will vary the strength of the prosodic edge cues available to aid children in locating word boundaries in fluent speech. Recall that a previous study in support of the Edge Hypothesis showed that 7.5-month-olds find it easier to segment utterance initial and final words from speech than utterance medial words [10]. Thus, we predict that infants’ earliest segmentation attempts at 6 months will be most successful when target words are flanked by major prosodic boundaries such as utterance edges. In line with past studies (e.g. [6]), we expect that 6-month-olds will fail to segment utterance medial words from speech, despite the presence of strong transitional-probability cues to word boundaries. Understanding when and under what circumstances infants first begin showing success in segmenting words from speech is crucial to providing an accurate account of early speech development. If 6-month-old infants were to show evidence of segmenting words from utterance edges earlier than they show evidence of segmenting words from utterance-medial position, then this finding would further underscore the importance of utterance level prosody in infants’ early word segmentation.

Finally, we report a corpus analysis examining the speech directed to or over-heard by a single 6-month-old for a period of three months. This input analysis, inspired by our four perceptual experiments, examines whether speech directed to the infant is more ideally suited to an edge-based segmentation strategy than child- or adult-directed speech. Importantly, we will not just examine how often words occur along utterance edges in the three registers, but also what types of words are most likely to occur in utterance initial and final position (e.g., proper names and pronouns), and whether frequent words are more likely to occur along utterance edges than infrequent words. By combining perceptual experiments with an in-depth analysis of the input to a young infant, we martial strong evidence in support of the Edge Hypothesis. That is, we show that thanks to the prevalence of utterance boundaries in infant-directed speech, word segmentation is not just something 6-month-olds accomplish under ideal circumstances. Rather, it is likely something they accomplish routinely. Moreover, our corpus analysis will provide support for the notion that learning how utterance edges sound could help infants learn many aspects of the language-specific sound structure of their native language, and thus aid in infants’ bootstrapping of language-specific cues to word boundaries. In the General Discussion, we conclude by discussing the potential impact of these findings for current models of developmental speech perception.

## Experiment 1: Utterance-edge versus utterance-medial position

Recent evidence suggests that word segmentation abilities may be present in infants earlier than 7.5 months. In this experiment, we use the Headturn Preference Paradigm of Jusczyk and Aslin [6] to ask whether 6-month-olds can segment words from natural speech only when target words occur in prosodically salient positions.

Six-month-olds were assigned to one of two conditions: the Edge-Aligned Condition or the Medial Condition. In the Edge-Aligned Condition, infants were familiarized with two passages, each containing a target novel word form alternating in utterance-initial and utterance-final position (e.g. ‘Geff runs the big circus in Toronto. It is such bad luck to have a rough Geff…’). In the Medial Condition, infants were familiarized with two passages, each containing a target word always placed in utterance-medial position (‘I like how Geff runs the circus. I wonder if Geff wants to juggle too….’). The test phase immediately followed the familiarization phase, and consisted of the presentation of isolated repetitions of words that either were or were not present in the familiarization passages. We predicted that if alignment with utterance boundaries facilitates segmentation in infants under 7.5 months of age, then participants should recognize familiarized word forms in the Edge Condition but not in the Medial Condition. In the context of this study, recognition would be reflected by a preference for listening to either familiarized or unfamiliarized targets during the test phase. An absence of a preference or either familiarized or unfamiliarized targets would lead to the conclusion that infants had not recognized the word forms.

### Method

All research carried out in this study was approved by the University of Toronto's Institutional Review Board. Written parental consent was obtained for each participating infant.

**Participants.** Fifty-six monolingual English-learning 6-month-olds from the Greater Toronto and Mississauga area were tested. Half of the infants were assigned to the *medial* group (12 females, 16 males; Mean age: 183 days; Range: 175–198 days) and half of the infants were assigned to the *edge-aligned* group (13 females, 15 males; Mean age: 181 days; Range: 164–197 days). The data from an additional 23 infants were discarded from analyses due to crying (17), falling asleep (1), average mean looking time differences greater than 2.5 standard deviations from the mean (2), and parental interference (3). As in all studies reported in this paper, infants who heard less than 90% English or who had reportedly recently suffered from an ear infection did not participate in the study. As a token of our appreciation for their participation, all participants in this study and all subsequent studies in this paper were given a junior scientist degree and either a lab t-shirt, a toy, or a book.

**Design.** Infants in the medial group were familiarized with two target words located in utterance-medial position and infants in the edge-aligned group were familiarized with two target words alternating between utterance-initial and utterance-final position. Within each group, an equal number of infants were randomly assigned to familiarization with one of the following consonant-vowel-consonant (CVC) word item pairs: cash and deeb, geff and pig, geff and cash, or deeb and pig. Note that all children were familiarized with one real English word (pig or cash) and one nonce word (geff or deeb). Since the two real words we used are not typically known by 6-month-olds, the target words were presumably all novel words from the infants’ perspective. The use of two real words and two nonce words as stimuli was simply a design constraint on the past studies for which these stimuli were originally recorded [10], [35]. During the test phase, all infants were presented with the same four test items: geff, cash, deeb, and pig. Thus, each test word item was familiar for one half of the infants and unfamiliar for the other half. Note that this counterbalancing is crucial to the design and the interpretation of the test phase results. All infants were exposed to the same test stimuli, but what was considered a familiar or unfamiliar word form was counterbalanced across infants. This ensures that any preferences for familiar or novel word forms observed in the test phase are not due to any inherent properties of the test items themselves. If a preference is observed for familiar or novel items then the only possible explanation for the differential responding to test items is the influence of the familiarization phase on infant behavior.

**Stimuli.** The speech materials were the same as those used in Seidl and Johnson [10], [35]. Stimuli were recorded by a female native English speaker naïve to the purpose of the study. She was instructed to produce the stimuli in a happy infant-directed register. The target words were first recorded in a sentence context, then in isolation. Each word was recorded in 18 sentences: six times in utterance-initial position, six times in utterance-medial position, and six times in utterance-final position (see the supporting information for sample passages; see Seidl & Johnson [10] for acoustic measurements). Sentences were concatenated into familiarization sound files, each consisting of six sentences with a given familiarization item occurring at the same sentence position in each file. Sentences were separated by brief silences (pauses were roughly the length of the speaker’s natural pause for breath between utterances). These six sentence passages were all roughly 20 seconds in duration. Isolated words were concatenated into four test lists, each containing 15 isolated repetitions of one of the four test items. Test list length ranged from 17.5 s to 17.9 s, with individual test items separated by approximately a half a second of silence (*M* = 503 ms, *SD* = 7 ms). Stimuli were presented at a comfortable listening volume.

**Procedure.** The same modified version of the Headturn Preference Procedure used by Jusczyk and Aslin [6] and Seidl and Johnson [10], [35] was used in the current experiment. Infants sat on their caregiver’s lap in the middle of a three-sided booth. The testing booth was located inside a double-walled IAC sound-attenuating booth, and was constructed of four-foot-high white pegboard panel. White curtains hung from the ceiling to a few inches below the top of the pegboard walls. A green light was located at eye level directly in front of the seated infant and red lights were located at the same height 90 degrees to the right and left of the infant. A speaker was hidden behind each of the red side lights. The experimenter sat outside of the sound-attenuating booth and watched the infant’s behaviour via a TV monitor. The experimenter relayed the infant’s looking behaviour to a computer via a button box. The presentation of flashing lights and sounds was computer-controlled. To prevent caregivers from influencing their infant’s looking behaviour, they listened to loud masking music over enclosed aviator-style headphones. Masking music was a mix of multiple simultaneous tracks of different speech stimuli from the experiment combined with music containing few pauses. As an additional precaution to prevent the introduction of caregiver bias, caregivers were kept naïve as to our specific looking time predictions until the completion of the study.

At the beginning of the experiment, the green center light would flash until the infant looked towards it. Once the infant looked towards the light, the experimenter would press the center button, at which point the center light turned off and one of the two red side lights began to flash. Once the infant looked to a flashing red side light, the experimenter indicated the direction of the infant’s gaze by pressing a button on the button box and a familiarization sound file began to play. The sound file continued until the infant looked away from the light for longer than 2 s, or the sound file came to the end. If the infant looked away for less than 2 s, then the sound file continued to play, but the time spent looking away from the light was not counted in the infant’s looking time. As soon as the audio ceased to play, the red side light turned off and the green center light began to flash. The familiarization period continued in this manner until the infant had accrued *at least* 45 s of *looking time* to each of the two familiarization passages. Thus, passages typically played for more than 90 seconds total because infants varied in how often and how long they looked away from the lights while the passages were playing.

The test immediately followed the familiarization phase. During the test phase the computer kept track of how long infants looked to the flashing side light during the presentation of each test list. The test phase consisted of 12 trials, grouped into three blocks of four trials. Each block contained two familiar item trials and two unfamiliar item trials. Familiar item test trials contained multiple tokens of a single familiarized item (e.g., geff, for a child who heard passages containing the words geff and pig during the test phase). Unfamiliar test item trials contained multiple tokens of a single unfamiliar item (e.g., deeb, for a child who heard passages containing the words geff and pig during the test phase). Each test item (e.g., pig or deeb) occurred once in each of the three blocks. The flashing of lights and presentation of sounds files was pseudo-randomized such that no more than three trials in a row could occur on the same side and no more than two sounds files containing the same familiarization item could occur in a row. The dependent measure was mean orientation time to one of the two side red lights during test file presentation. Importantly, throughout the entire experiment, the experimenter was blind to which sound file was being presented to the infant.

As in all experiments reported in this paper, after completing the experiment, and before looking at the data, the experimenter assigned all infants a fussiness rating on a scale of 1 to 5 (1 indicating an infant who appeared happy throughout the study and 5 indicating a baby who did not complete the study due to fussiness). Infants with fussiness ratings of 4 or 5 were excluded from further analyses.

### Results and Discussion

**Figure 1:**
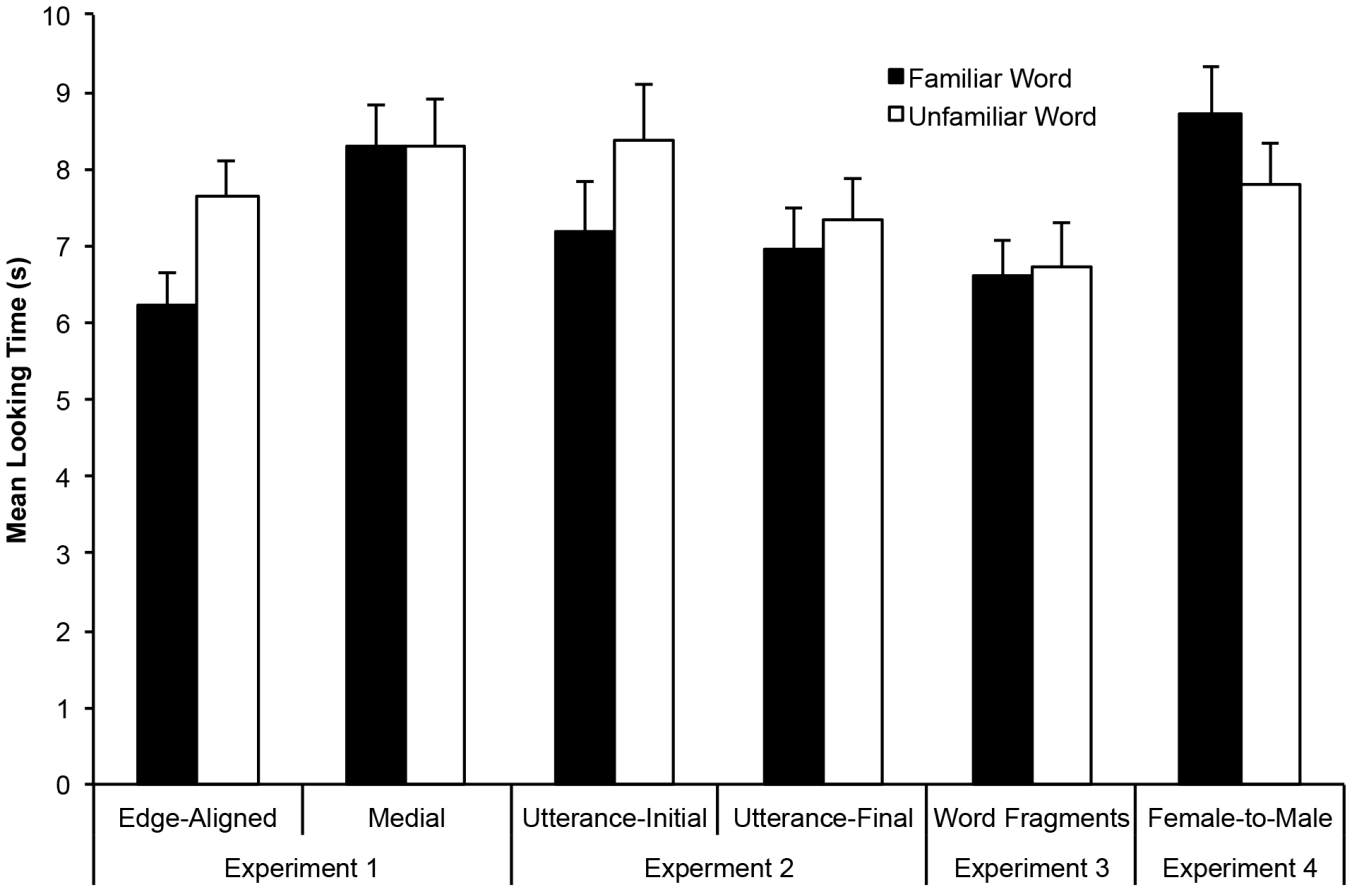
Results of Experiments 1-4. Mean looking time differences to familiar versus unfamiliar test word forms in Experiments 1 through 4. Six-month-olds successfully recognized familiarized word forms in Experiments 1, 2, and 4 (displaying a novelty effect in Experiments 1 and 2, and a familiarity effect in Experiment 4), but failed to show any recognition for the rhyme of the familiarized word forms in Experiment 3.

In the familiarization phase, the mean total length of time the passages played ranged from 112 to 188 s in the edge-aligned condition (*M* =  140 s, *SD* =  20 s) and from 110 to 190 s in the medial condition (*M* = 135 s, *SD* = 23 s). For the training phase, mean orientation times to the side light during familiar and unfamiliar trials were calculated for each infant (see Figure 1, panel A). Infants in the edge-aligned group oriented on average for 6.2 s (*SD* = 2.2 s) during the presentation of familiar item test trials and for 7.6 s (*SD* = 2.5 s) during the unfamiliar item test trials. Nineteen out of 28 infants in the edge-aligned group oriented longer towards the unfamiliar test items. Infants in the medial group oriented on average for 8.3 seconds (*SD* = 2.8 s) during the presentation of familiar item test trials and 8.3 seconds (*SD* = 3.3 s) during the unfamiliar item test trials. Eleven out of 28 infants in the medial group oriented longer towards the unfamiliar test item. A 2 (Item Location: medial vs. edge-aligned) x 2 (Test-Item Familiarity: familiar vs. unfamiliar) mixed design ANOVA revealed a significant main effect of item location, *F*(1, 54)  = 4.2, *p* = .045, and a significant effect of test-item familiarity, *F*(1, 54)  = 5.1, *p* = .03. Importantly, there was also a significant interaction between item location and test-item familiarity, *F*(1, 54)  = 5.4, *p* = .02. Two-tailed paired *t*-tests revealed that infants in the edge-aligned group oriented significantly longer to unfamiliar than to familiar test items, *t*(27)  = 3.4, *p* = .002, whereas infants in the medial group did not show a significant difference in their orientation times to familiar versus unfamiliar test items, *t*(27)  = 0.04, *p* = .97.

These results demonstrate that 6-month-old English learners can segment words from natural speech when those words are located along major prosodic boundaries. At the same time, data from the medial group replicate past studies with a more controlled stimulus set in that we fail to find any evidence that infants can segment words from utterance-medial position. Interestingly, the 6-month-old infants tested here demonstrated their recognition of words by longer orientation times towards unfamiliar rather than familiar words, rather than the opposite pattern as was observed in Jusczyk and Aslin [6]. Looking time preferences in word segmentation tasks often vary depending on the difficulty of the task and the perceptual distance between training and test items (e.g., [11], [35], [52], [53]). Although it is interesting to consider why infants shift between novelty and familiarity preferences, the direction of the preference does not affect the conclusion in this paradigm. Any consistent difference in looking times to familiar versus unfamiliar test items in the test phase indicates that the infants have detected a difference between the two types of test items. These results demonstrate that 6-month-olds can segment words from speech when the target words occur along utterance edges, but not when those same target words occur utterance-medially.

## Experiment 2: Alignment with one edge only

The results of Experiment 1 show that 6-month-olds segment words from speech when they are exposed to target items occurring multiple times at both the initial and final edge of an utterance, but not when items are presented utterance medially, which provides support for the Edge Hypothesis proposed in Seidl and Johnson [10] on the basis of data from 7.5-month-olds. The results of Experiment 1 are important because they represent the first demonstration that English-learning 6-month-olds can segment novel words from fluent natural speech even when the novel words are not flanked by a socially relevant well-known word such as the infant’s own name. However, by presenting the words in both utterance-initial and utterance-final position, we provided ample support for the segmentation task by providing infants with both the onset and offset of the target words flanked by silences. Therefore, in Experiment 2, we set out to test whether infants can segment words from speech if they only ever hear those words in utterance-initial or utterance-final position. In other words, can infants succeed at segmenting words from speech when they have only heard either the onset or offset of that word flanked by a major prosodic boundary? We see this as a more stringent test of 6-month-olds’ segmentation capabilities.

### Method

**Participants.** Forty monolingual English-learning 6-month-olds from the Greater Toronto and Mississauga area were tested (22 females; Mean age: 186 days; Range: 175–196 days). Half were assigned to the utterance-initial condition and half were assigned to the utterance-final condition. The data from an additional 13 infants were discarded from the analysis due to fussiness (12) or parental interference (1).

**Design, Stimuli, & Procedure.** Infants in the *utterance-initial* group were familiarized with two target items that were always located in utterance-initial position, and infants in the *utterance-final* group were familiarized with two target words that were always located in utterance-final position. In all other respects, the design of the experiment, the stimuli, and the procedure were identical to Experiment 1.

### Results and Discussion

Mean orientation times to the side lights while the passages were playing during familiarization ranged from 99 to 167 seconds (*M* =  133 s; *SD* = 20 s) in the utterance-initial group and from 102 to 165 seconds (*M* = 130 s, *SD* = 16 s) in the utterance-final group. For the training phase, mean orientation times to the side light during familiar and unfamiliar trials were calculated for each infant. Infants oriented on average for 7.1 s (*SD* = 2.7 s) during the presentation of familiar item test trials and 7.9 s (*SD* = 2.9 s) during the unfamiliar item test trials (see Figure 1, panel B). Twenty-six out of 40 infants listened longer to the unfamiliar test items than the familiar test items. A 2 (Item Location: utterance-initial vs. utterance-final) x 2 (Test-Item Familiarity: familiar vs. unfamiliar) mixed design ANOVA revealed no main effect of item location, *F*(1, 38)  = 0.6, *p* = .44, or interaction between item location and test-item familiarity, *F*(1, 38) = 1.1, *p* = .30. Importantly, however, the main effect of test-item familiarity was significant, *F*(1, 38)  = 4.6, *p* = .04. These results demonstrate that 6-month-old English learners can segment words from natural speech even if they have only heard either the onset or offset of the target word located along major prosodic boundaries. As in Experiment 1, infants demonstrated their recognition of words by orienting towards novel words for longer than they oriented to familiar words. In combination, Experiments 1 and 2 provide support for the Edge Hypothesis by demonstrating that 6-month-olds can extract words from fluent natural speech only when those words are flanked by an utterance boundary. In Experiment 3, we ask how robust these initial segmentation abilities really are.

## Experiment 3: Recognition of word fragments

The 6-month-olds tested in Experiments 1 and 2 succeeded in segmenting words from speech when those words were aligned with either the left or right edge of a major prosodic boundary. Since past research has shown that natural IDS consists of many short utterances (and *ipso facto* contains more word boundary-flanked words than ADS), then 6-month-olds’ word segmentation successes in the real world may be more common than previous research has suggested. We return to this issue in a corpus analysis reported at the end of this paper. However, before accepting the results of Experiments 1 and 2 as firm evidence that 6-month-olds readily segment utterance-boundary flanked words from speech, there is an alternative explanation for these results that we must consider. Perhaps infants have not segmented whole word-forms from speech, but simply responded to a salient repetitive sound in the familiarization passages, such as the vowel nucleus of the familiarized target words. Past studies have explored this issue by presenting infants with a near-familiar test word item that differs from the familiarized word by a single segment. The near-word test items in those studies were created by either changing the onset consonant (e.g., ‘tup’ instead of ‘cup’ in [6]; ‘pinome’ instead of ‘ginome’ in [54]) or deleting the onset consonant (e.g., ‘ice’ instead of ‘dice’ in [55]; ‘win’ instead of ‘twin’ in [56]) of the familiarized word. The logic underlying those studies is that if infants have segmented all details of the target word from speech (and not just a vague or incomplete representation of the word) they should show no recognition for the near-familiar word. In Experiment 3, we create test items that differ from familiar words by the deletion of the onset consonant (e.g., ‘eff’ instead of ‘geff). We refer to these near-familiar words as word fragments because they contain part but not all of a familiarized word.

In order to explore whether the 6-month-olds tested in Experiments 1 and 2 recognized familiarized words using a different strategy than slightly older infants we familiarized a new group of 6-month-olds with the same passages used in the edge-aligned condition of Experiment 1. We then tested these infants on the vowel nucleus and coda of the familiarized target words (e.g., ‘eff’ from ‘geff’). If infants were simply responding to the repetitive vowel sound in the familiarization passage (or the rhyme), then they should show the same orientation difference in the current experiment as they showed in the edge-aligned condition of Experiment 1.

### Method

**Participants.** Twenty-four monolingual English-learning 6-month-olds from the Greater Toronto and Mississauga area were tested (11 females, 13 males; Mean age: 186 days; Range: 173–198 days). The data from an additional 7 infants were discarded from the analysis due to fussiness.

**Design.** The design of Experiment 3 was identical to the edge-aligned condition of Experiment 1. The only difference was that during the test phase, all infants were tested on the word fragments ig, eff, ash, and eeb rather than pig, geff, cash, and deeb.

**Stimuli.** The passages used were the same as those used in Experiment 1. The word-fragment test items were recorded by the same speaker who produced the passages and words in isolation from Experiment 1. Test list length ranged from 15.0 s to 15.9 s, with individual test items separated by approximately a half a second of silence (*M* = 585 ms, *SD* = 9 ms).

**Procedure.** The same procedure was used as in Experiment 1.

### Results and Discussion

In the familiarization phase, mean orientation times to the side lights while the passages were playing ranged from 110 to 183 seconds (*M* = 134, *SD* = 17 s). For the training phase, mean orientation times to the side light during familiar (word fragment) and unfamiliar (word fragment) trials were calculated for each infant. Infants oriented, on average, for 6.6 s (*SD* = 2.2 s) during the presentation of familiar target word test trials and for 6.7 s (*SD* = 2.8 s) during the unfamiliar target word test trials. Twelve out of 24 infants looked longer during unfamiliar test trials than familiar test trials. A two-tailed paired *t*-test revealed no significant effect of test-item familiarity, *t*(23)  = 0.26, *p* = .80. A 2 (Test-Item Familiarity: familiar vs. unfamiliar) x 2 (Experiment: 1 vs. 3) mixed-design ANOVA comparing the results of the edge-aligned condition of Experiment 1 and the current experiment revealed a significant main effect of item familiarity, *F*(1, 50)  = 6.0, *p* = .02, and a significant interaction between Experiment and Test-Item Familiarity, *F*(1, 50)  = 4.2, *p*  = .045. No main effect of Experiment was observed, *F*(1, 50)  = 0.2, *p* = .66.

Thus, the 6-month-olds in Experiment 3 and the infants tested in the edge-aligned condition of Experiment 1 behaved differently, as the infants in the current experiment did not treat the nucleus and coda of the familiarized word forms as familiar. These results suggest that the 6-month-old English learners tested in Experiments 1 and 2 were segmenting out entire word forms, and not just a salient portion of the word form such as the vocalic nucleus or rhyme. These results support the notion that the ability to segment words from speech may begin earlier and in a more robust fashion than earlier studies have suggested.

## Experiment 4: Recognition across acoustically distinct realizations

In combination, Experiments 1 through 3 suggest that 6-month-olds can segment words from speech, as long as those words are aligned with an utterance boundary. This finding fits well with reports that by 6 to 9 months of age, infants have started to segment words from speech in certain contexts (e.g., [28], [46]) and are already beginning to build a rudimentary lexicon [49]–[51]. Thus, we argue that our findings suggest that English-learning 6-month-olds may be better positioned to segment words from their speech input than past studies have suggested. However, one could argue that we presented infants with an over-simplified segmentation task in Experiments 1 and 2, or that the infants only show evidence for segmenting the edge-flanked words from speech because the edge-flanked words are acoustically similar to our isolated word test items. That is, 6-month-olds may only show recognition of familiarized word forms when the tokens produced in familiarization and test phase are close acoustic matches. If this were the case, then the impressive segmentation performance we have observed in Experiments 1 and 2 may not reflect the capabilities of 6-month-olds in their everyday environments. In order to make efficient use of all of their language input, infants would need to recognize words across considerable acoustic-phonetic variation, including speaker-related variation.

A recent study has shown that 7.5-month-olds can recognize the mapping between word tokens produced in a male and female voice if the infants are initially familiarized with fluent passages containing the words and then tested on isolated words [57]. Even though 7.5-month-olds can succeed at this task, other studies suggest that mapping highly acoustically distinct tokens of a word onto the same underlying word representation is more difficult for 7.5- to 9-month-olds than mapping acoustically similar tokens of a word onto the same underlying representation (e.g., [11], [58], [59]). Thus, in Experiment 4, we further tested the limits of English-learning 6-month-olds’ segmentation capabilities by presenting infants with the familiarization passages from Experiment 1, recorded in a female voice, and then presenting the test items in a male voice. If 6-month-olds are like 7.5-month-olds, in that they readily extract robust representations of the word forms from speech, then the infants in Experiment 4 should be able to recognize the familiarized words in the test phase despite the speaker change.

### Method

**Participants.** Twenty-eight monolingual English-learning 6-month-olds from the Greater Toronto and Mississauga area were tested (15 females, 13 males; Mean age: 183 days; Range: 160–196 days). The data from an additional 7 infants were discarded from the analysis due to fussiness (6) or experimenter error (1).

**Design.** The design of Experiment 4 was identical to the edge-aligned condition of Experiment 1. The only difference was that the test items were produced by a male voice rather than the same female voice that had produced the familiarization passages.

**Stimuli.** The familiarization passages used were the same as those used in the edge-aligned condition of Experiment 1. The test items, however, were recorded in IDS by a new male speaker. Five isolated repetitions of each of the target words from Experiment 1 were recorded. In each test list, the five tokens of a single item were combined into a block that was presented three times, so that each test list contained 15 repetitions of a single target word, each separated by approximately 500 ms of silence.

**Procedure.** The same procedure was used as in Experiment 1. The only difference was that the presentation of the familiarization passages during the first part of the experiment was not contingent upon infants’ looks to the side lights. Rather, a 90-s recording of the two alternating passages played as soon as infants looked for the first time to the center blinking light. During this 90 s familiarization phase, infants were exposed to *exactly* 12 tokens of each of the two target words (note that infants in Experiments 1 through 3 were exposed to a few more tokens of at least one target word because with the contingent exposure paradigm they had to accrue *at least* 45 seconds of looking time to each of two target word passages before the test phase began). The lights continued to turn on and off contingent upon infants’ looking behaviour throughout the familiarization phase, however the sound played continuously. This modification to the procedure used in Experiment 1 and 2 was made because we have found that it reduces the length of the experiment and thus reduces the likelihood that infants will become fussy before the test phase begins. This sort of procedure has been used in many infant segmentation experiments in the past (e.g., [22], [57]) and performance does not appear to differ from that in the orientation-contingent familiarization procedure used in Experiments 1 and 2.

### Results and Discussion

Mean orientation times to the side light during familiar and unfamiliar trials were calculated for each infant. Infants oriented, on average, for 8.7 s (*SD* = 3.1 s) during the presentation of familiar-item test trials and 7.8 s (*SD* = 2.7 s) during the unfamiliar-item test trials (see Figure 1, panel C). Twenty out of 28 infants looked longer during familiar-item trials than unfamiliar-item trials. A two-tailed paired *t*-test revealed a significant effect of test-item familiarity, *t*(27)  = 2.51, *p* = .02, driven by significantly longer looking times during familiar trials than during unfamiliar trials. The direction of this preference differs from Experiment 1, but this is not entirely unexpected given that longer looking times to familiar than unfamiliar items have been reported in cross-gender word-recognition studies [57], [58]. As a segmentation task becomes more difficult, and/or the acoustic distance between training and test items increases, it is common for infants to shift from a novelty preference to a familiarity preference (e.g., see [52], [53], [60], [61]), for similar directional shifts in preference associated with task difficulty). In summary, the results demonstrate that infants successfully segmented the target words from the fluent passages heard during the familiarization phase in spite of the change of speaker and that 6-month-olds possess remarkably robust word segmentation skills.

## Corpus Study

Recall that an important prediction of the Edge Hypothesis, as proposed in Seidl and Johnson [10], is that infants should be particularly attentive to utterance edges, and initially find it easier to segment words from utterance edges than from utterance-medial position. In combination, the four experiments we have reported thus far provide strong support for this hypothesis. English-learning 6-month-olds segmented words from natural speech as long as those words were flanked by at least one utterance boundary (Experiments 1 and 2). Further, the word form representations that 6-month-olds extract from speech are robust and whole (Experiment 3) as well as speaker independent (Experiment 4). Now that we have demonstrated that English-learning infants begin segmenting words from natural speech by as early as 6 months of age (even when target words are not flanked by a highly familiar proper name, as they were in [46]), we turn to ask what implications these findings have for contemporary theories of developmental speech perception. How effective could attention to utterance edges be for supporting infants’ first word segmentation attempts? If attention to utterance edges is important, one might expect to find that IDS is particularly well suited for the application of such a segmentation strategy. That is, one would expect that not only should utterance boundaries be highly prevalent in IDS, there should also be a wide range of word types occurring along utterance edges. Moreover, it would be useful if the word types that occur along edges tended to be highly frequent so that infants would have a better chance at extracting them for speech and storing them in memory.

There is ample evidence that, in comparison to ADS, IDS consists of shorter, more prosodically exaggerated utterances (e.g., [62], [63]). Language input consisting of many short utterances must, therefore, contain more words at utterance-boundaries than ADS. Indeed, a recent analysis of utterances spoken to 9- to 15-month-old English-learning children demonstrates that utterance boundaries are common in speech directed to this age group [47]. Would this also be the case for speech directed to younger infants, such as the 6-month-olds tested in the current study? And what types of words occur along utterance edges? Would the words occurring along utterance edges belong predominantly to any particular grammatical class? Would the most frequently occurring words be more likely to occur at utterance edges than less frequent words? Addressing these questions represents an important step in testing the viability of the Edge Hypothesis in explaining infants’ first steps in word segmentation and in subsequent word learning. In the remainder of this section, we compare everyday language interchanges in infant-, child-, and adult-directed speech with the goal of determining whether the positioning of words in infant- (and possibly also child-) directed speech is particularly well suited for the application of an edge-based segmentation strategy.

We base our analysis on the van der Weijer corpus [18], which is comprised of recordings of all the speech heard by a single Dutch- and German-learning infant beginning at the age of 6 months 0 days and recorded for 91 days. Although it may have been preferable to select an English corpus for comparison with Experiments 1–4, the strength of this corpus is that it represents a dense sample of naturalistic input provided to a young infant similar in age to the infants we tested in those experiments and, as far as we are aware, no such publicly available corpus exists for an English-learning infant. The transcribed corpus consists of a sample of 18 days from that period – seven from the beginning (days 1–7), six from the middle (days 39–44), and five from the end (days 86–90). Van de Weijer [18] excluded utterances from his analyses that contained babytalk and routines, such as nursery rhymes and songs. Also excluded was any utterance that contained an untranscribable word. For our analyses we included all utterances and word types, so our statistics may deviate slightly from those reported in van de Weijer. It should also be noted that, unlike Swingley [23], we included both the mother’s Dutch and German utterances in our analyses. The corpus also contains transcriptions of utterances directed towards the infant’s older sibling, aged 2 yrs 6 months to 2 yrs 9 months, and of the adult-to-adult interactions between the mother, father, and babysitter. Thus, we were able to compare infant- (IDS), child/toddler- (CDS), and adult-directed speech (ADS). Our analyses included 16242 IDS utterances, 43773 CDS utterances, and 21377 ADS utterances.

To test whether infant-directed input would support an edge-based segmentation strategy, we first compared the length of utterances in IDS, CDS, and ADS, and the proportion of word tokens in each register that occurred in isolation, were flanked by at least one utterance boundary, or occurred in the middle of an utterance. Consistent with van de Weijer [18], mean length of utterance (MLU) differed as a function of register, *F*(2, 81389)  = 1986.36, *p*<.001, η_p_^2^ = .05. On average, infant-directed utterances were 2.65 words long, child-directed utterances were 3.10 words long, and adult-directed utterances were 4.42 words long. Post-hoc comparisons using Tukey’s HSD showed that all three MLUs differed significantly from each other (all *p*s <.001).

For the remainder of our analyses we computed the position of each word token in each utterance and tallied whether it occurred in isolation, at an utterance edge (beginning or end), or in the middle. Note that words occurring in isolation are in fact just words flanked by two edges. Nonetheless, we report words occurring in isolation separately for completeness sake, and also because the rate of occurrence of isolated words in IDS has been such an important issue in past studies (e.g., [20], [64]). We excluded words that were not transcribable from the analysis (IDS: 1219 words, CDS: 4979 words, ADS: 4766 words), but included any other words occurring in the same utterance. The analyses were therefore based on the following data; IDS: 2012 word types across 41778 tokens, CDS: 4398 word types across 130437 tokens, ADS: 5423 word types across 89544 tokens.

As the three corpora differ in size, it is necessary to use a normalized measure when comparing across registers. We therefore converted the raw frequencies of each word type in each utterance position to word frequency per million. It should be noted that the differences in corpus size might affect the frequency estimates because larger corpora are more likely to capture every low frequency word, thus lowering the overall mean word frequency. Therefore, care should be taken in interpreting absolute frequency differences across the three corpora in this analysis. Our focus, rather, is on the relative frequencies of occurrence of the same word types in different utterance positions. For an analysis of word repetition controlling for sample size and input language, see van de Weijer [18]. In addition to relative word frequencies across utterance position, we also report the relative proportion of word tokens in each utterance position.

**Figure 2:**
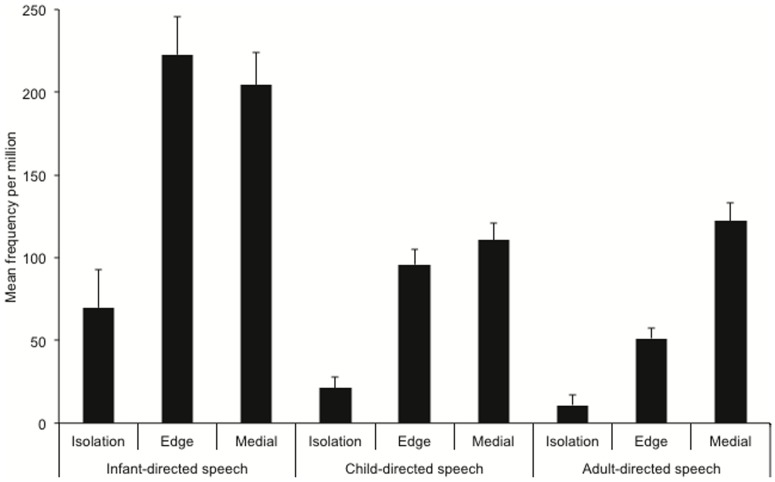
Frequency of word occurrence per million broken down by utterance position and speech register.

First, we examined the average word frequency per million, broken down by speech register and positioning with respect to an utterance boundary. We converted the raw frequency to frequency per million for each word, in each utterance position, in each register, and analysed the data using a mixed ANOVA with planned contrasts. There were two non-orthogonal between-group contrasts, one comparing IDS with CDS, and the other comparing CDS with ADS. One within-item contrast compared words flanked by at least one utterance boundary (words in isolation versus those at the edge of a multi-word utterance) with words occurring utterance medially, and another compared words in isolation with words at the edge of multi-word utterances. To compensate for the non-orthogonal contrasts, we used a Bonferroni-adjusted alpha level of.01875. The results are presented in Figure 2. Overall word frequency (and, hence, repetition of individual words) was significantly higher in IDS than CDS, *F*(1,11830)  = 40.40, *p*<.001, but there was no difference in average word frequency for CDS versus ADS, *F*(1,11830)  = 1.83. Across all speech registers, word frequency was significantly lower at utterance edges than utterance medially, *F*(1,11830)  = 68.73, *p*<.001. Thus, words occur more frequently utterance medially than utterance finally, which is to be expected given that there are usually more medial than edge positions available in an utterance. This interacted with the CDS versus ADS contrast, *F*(1,11830)  = 5.68, *p* = .017, such that the difference in word frequency between words at utterance edges and words occurring word medially was more pronounced for ADS than CDS, but there was no interaction with IDS versus CDS. This can be attributed directly to the MLU – as the mean utterance length increases there are simply more words occurring utterance-medially.

Considering now only words occurring at utterance edges, words occurred significantly less frequently in isolation, on average, than at the edge of multi-word utterances, *F*(1,11830)  = 263.43, *p*<.001, and this interacted significantly with IDS versus CDS, *F*(1,11830)  = 28.16, *p*<.001, and with CDS versus ADS, *F*(1,11830)  = 9.56, *p*  = .002 (see Figure 2). These results show that words at utterance edges occur relatively more frequently at the edge of a multi-word utterance than they do in isolation, and this difference is more pronounced in IDS than CDS, and in CDS than ADS. Together, these findings demonstrate that words are more available at utterance edges, particularly in multi-word utterances, in IDS than in the other registers.

Having now demonstrated that the short MLU of IDS, coupled with a more restricted input vocabulary than CDS and ADS, results in high repetition of words at utterance edges, it could be argued that an edge-based strategy may not be as useful to infants as it could be because certain grammatical categories are likely to be more prevalent at edges than others. If, for example, the majority of words occurring at utterance edges are closed-class function words, then the sound structure of these items might not reflect the typical sound structure of content words (e.g., [65]). Thus, our second analysis reports both word frequency and the relative proportion of word locations, split by grammatical category. As can be seen in Table 1, determiners, prepositions, and conjunctions are relatively infrequent in IDS, in any position (< 20 per million overall), but for all other grammatical categories (> 30 per million overall) the percentage of occurrences in isolation or utterance-medially ranged from 31% to 85%. This indicates that if infants began segmenting words from speech by focusing on edge-aligned words, they would have access to a wide variety of word forms.

**Table 1:** Frequency per million of words occurring in isolation, at the edge (beginning or end), or in the middle of an utterance, split by grammatical category for infant- (IDS), child- (CDS), and adult-directed speech (ADS).

		IDS			CDS			ADS		
Grammatical Category		Isolation	Edge	Medial	Isolation	Edge	Medial	Isolation	Edge	Medial
Interjection	Frequency	56	67	21	14	17	3	9	11	4
	*Percent*	*39*	*46*	*15*	*42*	*50*	*8*	*38*	*45*	*17*
Adverb	Frequency	2	28	40	2	18	25	0	9	25
	*Percent*	*2*	*40*	*57*	*4*	*39*	*57*	*1*	*26*	*73*
Pronoun	Frequency	0	19	33	1	7	4	1	8	19
	*Percent*	*1*	*36*	*63*	*8*	*59*	*33*	*2*	*29*	*69*
Verb	Frequency	2	28	19	1	17	13	0	5	13
	*Percent*	*4*	*57*	*39*	*5*	*55*	*40*	*1*	*29*	*71*
Noun	Frequency	5	25	17	1	11	12	0	5	13
	*Percent*	*10*	*54*	*36*	*4*	*46*	*50*	*1*	*26*	*73*
Auxiliary Verb	Frequency	0	10	23	0	4	10	0	2	12
	*Percent*	*0*	*31*	*69*	*0*	*26*	*74*	*0*	*17*	*83*
Proper Noun	Frequency	4	23	6	1	8	15	0	1	2
	*Percent*	*11*	*71*	*18*	*3*	*35*	*63*	*3*	*37*	*59*
Adjective	Frequency	1	14	15	1	5	5	0	3	8
	*Percent*	*4*	*47*	*48*	*8*	*46*	*46*	*3*	*27*	*70*
Determiner	Frequency	0	2	17	0	2	12	0	1	11
	*Percent*	*0*	*12*	*88*	*1*	*14*	*86*	*0*	*10*	*90*
Preposition	Frequency	0	4	13	0	4	9	0	2	11
	*Percent*	*2*	*24*	*74*	*1*	*30*	*69*	*0*	*15*	*85*
Conjunction	Frequency	0	2	1	0	2	2	0	4	5
	*Percent*	*0*	*55*	*45*	*0*	*61*	*39*	*1*	*43*	*56*
Total	Frequency	70	223	205	21	96	110	11	51	122
	*Percent*	*14*	*45*	*41*	*9*	*42*	*49*	*6*	*28*	*66*
Total Minus Interjections	Frequency	14	156	183	7	78	108	2	41	118
	*Percent*	*4*	*44*	*52*	*3*	*41*	*56*	*1*	*25*	*74*

The relative proportions of words in each utterance position in each speech register are indicated as a percentage underneath the frequency per million. Grammatical categories are ordered according to overall word frequency in IDS, from highest to lowest. Frequency per million has been rounded to the nearest whole integer.

One surprise to emerge from this analysis is the large proportion of words in IDS that were coded as interjections (29%; e.g., *ja*, *zo*, *he*, *hai*). It also appears from the overall relative proportions of words in each utterance position that there is a diminishing importance of words in isolation from IDS through CDS to ADS. However, after removal of the interjections from the total (see Table 1) it can be seen that words are distributed similarly for IDS and CDS, but both have a greater proportion of words occurring at utterance edges than in ADS. Two important factors to note here are that IDS is distinct from both CDS and ADS, suggesting that corpus work examining the plausibility of early segmentation strategies should focus on input directed towards the appropriately aged infant (as we have done in the current analysis). In addition, the inclusion or exclusion of interjections dramatically alters how we characterize IDS. We return to the latter point in the General Discussion.

Our first analysis showed that although words occur most frequently in medial position, overall, they occur relatively more frequently at utterance edges in IDS and CDS versus ADS. This is attributable to the shorter length of IDS and CDS utterances, relative to ADS. The analysis of grammatical categories showed that a wide variety of word types are available in IDS and CDS at utterance edges and, therefore, an edge-based strategy would be useful for infant word learning. Here we examined whether those word types that are more frequent in the infant’s input are more likely to occur at utterance edges than in medial position as compared to those that are less frequent in the input. We reasoned that if certain words occurring in utterance aligned position were particularly repetitive in IDS, then this could further facilitate infants’ early segmentation. For this analysis, we compared the 50 most frequent noun types in the input to all other nouns types (*n* = 607; see Table 2). We focus this analysis on nouns because nouns are thought to be one of the earliest word classes infants segment from speech [e.g., 66] and they have been the focus of most infant word segmentation studies [e.g., 6], including the current study. Our analysis reveals that the proportion of frequent nouns occurring in isolation or in utterance-edge position in IDS is much higher than medial position (73% vs. 27%), but for less-frequent nouns the proportion is roughly the same (52% vs. 48%). The proportions are roughly equal in CDS for both frequent and less-frequent nouns, and there is also no difference in the proportions across frequent and less-frequent nouns in ADS, although nouns in the adult register occur mostly utterance medially. Here we find that IDS patterns differently than both CDS and ADS when it comes to highly frequent nouns. Attending to the edges of utterances in IDS would present the opportunity for infants to learn highly repetitive words, and this may help them solve the word segmentation problem.

**Table 2:** Frequency per million of nouns occurring in isolation, at the edge (beginning or end), or in the middle of an utterance, for infant- (IDS), child- (CDS), and adult-directed speech (ADS), split by the top 50 nouns in the child’s input versus the remainder of the nouns.

		Top 50 nouns			All but the top 50 nouns		
Speech Register		Isolation	Edge	Middle	Isolation	Edge	Middle
IDS	Frequency	134	624	277	1	10	10
	*Percent*	*13*	*60*	*27*	*6*	*46*	*48*
CDS	Frequency	17	371	406	1	7	8
	*Percent*	*2*	*47*	*51*	*5*	*46*	*49*
ADS	Frequency	3	139	444	0	3	9
	*Percent*	*1*	*24*	*76*	*1*	*27*	*71*

The relative proportions of noun tokens in each utterance position in each register are indicated as a percentage underneath the frequency count. Frequency per million has been rounded to the nearest whole integer.

## General Discussion

A large number of studies have shown that English-learning infants have started solving the word segmentation problem well before their first birthday, but the details of how and when they begin to accomplish this feat are not yet clear. A good strategy for understanding how segmentation abilities develop is to better understand the scope and nature of infants’ very first segmentation attempts. Past work in this area has focused largely on infants 7.5 months and older, but this age group is already using multiple language-specific cues to segment words from speech, and the question remains how infants initially bootstrap this information from the input. In this study, we have demonstrated that attention to utterance edges allows young infants to segment words from fluent natural speech earlier than past studies have suggested. More specifically, we have shown that infants are able to segment words at utterance-edges from speech by 6 months of age, well before English-learning infants demonstrate any ability segment utterance-medial words from natural speech. Our findings fit well with recent reports in the literature regarding the precocious nature of infants’ word segmentation and recognition abilities [28], [49]–[51], and can also be seen as support for the Edge Hypothesis, as proposed in Seidl and Johnson [10], [35].

In Experiments 1, 2, and 4 we presented 6-month-olds with a set of progressively more difficult segmentation tasks. Remarkably, infants succeeded in even the most difficult task. Most notably, in Experiment 4, we show that from as early as 6 months of age, infants can already map tokens of the same word spoken by different speakers onto the same underlying abstract representation. This is impressive, as previous studies have shown that even 7.5-month-olds find it difficult to cope with speaker-related variation in a speech segmentation task (e.g., [58]; see also [11], for a related discussion]. Importantly, as demonstrated by Experiment 3, 6-month-olds’ ability to cope with speaker variation is not due to an inability to form a detailed representation of the familiarized words. When infants were presented with just the rhyme of the familiarized target words, they showed no recognition, suggesting they had extracted the whole CVC word from speech. In combination, the results of Experiments 3 and 4 fit well with both classic and contemporary reports in the literature demonstrating that very young infants handle indexical and phonological variation in a surprisingly sophisticated manner (e.g., [57], [67], [68]).

Our corpus analysis was inspired by the four perceptual experiments reported in this paper. In our analysis, we found convergent evidence for the usefulness of the Edge Hypothesis, suggesting that IDS provides suitable input for infants to benefit from attending to the edges of utterances. According to the Edge Hypothesis, the edges of major prosodic boundaries serve as hotspots for early segmentation attempts before infants have yet learned any language-specific word segmentation heuristics such as the prevalence of word initial stress in English. According to this hypothesis, utterance edges not only offer statistically reliable cues to word boundaries, but also provide the infant with perceptually salient boundaries (for discussion, see [10], [36]). If infants use an edge-based segmentation strategy, then word segmentation would be facilitated if a relatively large proportion of words in IDS were flanked by an utterance boundary, and if the words occurring along utterance edges were particularly repetitive in IDS, thereby making it even easier for infants to acquire these words. Our corpus analysis reveals that both of these conditions are in fact true, indicating that IDS is well suited for the application of an edge-based segmentation strategy. Indeed, although the utterance lengths used in the four perceptual studies we report in this paper were between 7 and 10 words, the typical utterance length of infant-directed speech in our corpus analysis was between 2 and 3 words. This not only shows that attention to utterance boundaries could be an extremely useful segmentation strategy for young infants, but it also suggests that the segmentation task we presented infants with in our experiments was, at least in terms of utterance length, more difficult than the segmentation task they face in their everyday home environment when speech is directed to them.

As mentioned earlier, an unexpected additional observation from the corpus analysis was the overwhelming prevalence of interjections in IDS. In English, interjections include words such as ‘oh no’, ‘wow’, ‘oops’, and ‘ok’. These words carry no clear lexical meaning, nor do they perform the same sort of grammatical function such as content (e.g. nouns and verbs) and grammatical words (e.g. pronouns and determiners). One could argue that such word types might not be important for the very fact that they typically do not serve a clear grammatical function, however, we chose to include these items in our analyses for several reasons. First, interjections are far more frequent in IDS than CDS or ADS. Indeed, in our analysis, interjections account for 29% of the words in utterances addressed to infants. Second, they account for 80% of the words infants hear uttered in isolation, suggesting that these words would be highly accessible to infants. And third, it would seem that infants initially have no way of distinguishing between a content word, a grammatical word, and an interjection, so it seems odd to exclude a class of words that infants are likely attending to as potentially important in the input. Even if these words do not carry a clear meaning, they definitely carry important emotional messages as well as information about prosodic structure.

As a consequence of including interjections in our analysis, we observed a greater proportion of one-word utterances in IDS than ADS. However, this difference disappeared when we removed all interjections from our analysis. Thus, interjections are not only highly frequent, they also occur frequently in isolation. Past research on word learning in young infants has focused on early learning of word forms with concrete meanings (e.g., [49]–[51]) or clear grammatical functions (e.g., [69]). But given the suggestion in our analysis that some of the most frequent words an infant hears may be interjections, it may be the case that interjections play a far more important role in early language development than previously thought. Indeed, they might help children to break up multi-word utterances since we also found that they occur frequently there.

Since utterance boundaries necessarily align with word boundaries, another important corollary of the Edge Hypothesis is that attention to the edges of major prosodic boundaries may help infants learn language-specific segmentation strategies. For example, children might be able to use this information to help them learn that words tend to carry word-initial stress in English, and that they rarely start or end in certain phoneme sequences. Although we have not specifically demonstrated this in the current study, we do believe there is some evidence that this may be the case. For example, adults learn phonotactic rules better when they occur at the ends of utterances [48], and adults can even learn the phonotactic patterns defining word boundaries from an artificial language containing no cues to word boundaries other than utterance boundaries [70]. Moreover, it has been argued that German- and French-learning infants learn language-specific cues to word boundaries before they segment words from speech [32]. Perhaps attention to utterance edges helped these infants acquire this information. Added support for the notion that infants may be able to bootstrap at least some language-specific segmentation cues from utterance edges comes from our corpus analysis that revealed that the words occurring at utterance edges are not largely limited to a certain phonologically distinctive word class, such as function words. Thus, attention to utterance edges may very well begin to provide young children with a good approximation of how words in the language typically sound. Examining children’s use of utterance edges to learn language-specific segmentation strategies will be an interesting avenue to pursue in the future.

In sum, the four experiments and corpus analysis presented in this study provide compelling evidence for the Edge Hypothesis. Six-month-olds readily segment words at utterance edges, and IDS contains many repetitive words in precisely these locations. However, besides the fact that our analysis only looked at the input to a single child, another limitation of the current study is that we focused solely on words located along utterance edges (the same position we focused on in our behavioural experiments). In naturalistic language input, in contrast, utterances are further broken down into perceptually salient smaller component units including Intonational and Phonological Phrases. Even newborns readily perceive prosodic boundaries above the Phonological Phrase [38], [39], and 6-month-olds appear to take this information into consideration when segmenting words from an artificial language [28]. Thus, infants may be able to use phrasal (rather than just utterance) boundaries to find even more word boundaries than our corpus analysis suggests. In the future, it would be useful to further examine this issue with both behavioural studies as well as additional prosody-focused corpus analyses.

The goal of the four experiments reported in this study was not simply to confirm our intuition that young infants can segment words from speech as early as 6 months of age or that infants in general would find words positioned along utterance edges easier to extract from fluent speech than words positioned utterance medially. Indeed, 6-month-olds have already been shown to segment words from speech in certain situations (e.g., [46]) and 7.5-month-olds have already been shown to segment words from utterance edges more efficiently than they segment words from utterance-medial position [10]. Rather, we sought to show that word segmentation at 6 months of age (prior to 7.5 months of age, the age English learners acquire stress-based segmentation strategies) is not just possible in exceptional circumstances (when, for example, a target word is preceded by the infant's own name, [46]), but a common occurrence. Moreover, we sought to show that prior to the acquisition of language-specific sound structure cues to word boundaries (e.g. lexical stress and phonotactic information), transitional probabilities between syllables are not the only important factor determining infants’ word segmentation success.

Our findings also demonstrate that, at this early age, it is not necessary to suggest that all language-specific segmentation strategies are necessarily derived from an initial transitional probability tracking strategy (e.g., a possibility raised by [19], [24]). Despite the fact that all target items in Experiments 1 to 4 were flanked by strong TP cues to word boundaries, only those words that were also positioned along an utterance edge were segmented from speech. Clearly, utterance edges play a crucial role in infants’ early segmentation attempts, and any model of early speech perception that does not incorporate this fact will present a distorted view of early word segmentation abilities.

In conclusion, although it is clear that word segmentation abilities begin to develop in infancy, what is less clear is precisely how infants first begin to segment words from speech, as each language in the world is characterized by a unique constellation of word segmentation cues that can only be acquired through experience with that specific language. Past research has suggested that infants might begin to solve the word segmentation problem by tracking transitional probabilities between syllables (e.g., [19], [24]). Evidence for this view comes from studies showing that 6-month-olds can use syllable transitions, but not lexical stress, to extract words from an artificial language. In the current study, we have offered an additional, or perhaps even alternative strategy that young infants could use to begin segmenting words from speech. The current study was not designed to determine the relative importance of these two segmentation strategies, however, this is an important question to be considered in future research.

## Supporting Information

List S1 **Sample familiarization passages with alternating utterance-initial and utterance-final target words (Experiment 1, 3, and 4).**
(DOC)

## References

[1] Cole RA, Jakimik J (1980) A model of speech perception. In R. A. Cole (Eds.), Perception and production of fluent speech. Hillsdale, NJ: Erlbaum. pp. 133–163

[2] Endress AD, Hauser MD (2010) Word segmentation with universal prosodic cues. Cognitive Psychology 61: 177–199. 20573342 10.1016/j.cogpsych.2010.05.001

[3] Cutler A (1994) Segmentation problems, rhythmic solutions. Lingua 92 81–104.

[4] Cutler A (2012) Native listening: Language experience and the recognition of spoken words. Cambridge, MA: The MIT Press.

[5] Tyler MD, Cutler A (2009) Cross-language differences in cue use for speech segmentation. Journal of the Acoustical Society of America 126: 367–376. 19603893 10.1121/1.3129127 PMC2723901

[6] Jusczyk PW, Aslin RN (1995) Infants′ detection of the sound patterns of words in fluent speech. Cognitive Psychology 29: 1–23. 7641524 10.1006/cogp.1995.1010

[7] Jusczyk PW, Hohne EA (1997) Infants’ memory for spoken words. Science 277: 198. 10.1126/science.277.5334.1984 9302291

[8] Jusczyk PW, Houston DM, Newsome M (1999) The beginnings of word segmentation in English-learning infants. Cognitive Psychology 39: 159–207. 10631011 10.1006/cogp.1999.0716

[9] Mattys SL, Jusczyk PW (2001) Phonotactic cues for segmentation of fluent speech by infants. Cognition 78: 91–121. 11074247 10.1016/s0010-0277(00)00109-8

[10] Seidl AH, Johnson EK (2006) Infant word segmentation revisited: Edge alignment facilitates target extraction. Developmental Science 9: 565–573. 17059453 10.1111/j.1467-7687.2006.00534.x

[11] Singh L, Morgan JL, White KS (2004) Preference and processing: The role of speech affect in early spoken word recognition. Journal of Memory and Language 51: 173–189.

[12] Cutler A, Carter DM (1987) The predominance of strong initial syllables in the English vocabulary. Computer, Speech, and Language 2: 133–142.

[13] Cutler A, Norris D (1988) The role of strong syllables in segmentation for lexical access. Journal of Experimental Psychology: Human Perception and Performance 14: 113–121.

[14] Jusczyk PW, Cutler A, Redanz N (1993) Preference for the predominant stress patterns of English words. Child Development 64: 675–687. 8339688

[15] Houston DM, Jusczyk PW, Kuijpers C, Coolen R, Cutler A (2000) Both Dutch and English-learning 9-month-olds segment Dutch words from fluent speech. Psychonomic Bulletin & Review 7: 504–509. 11082857 10.3758/bf03214363

[16] Mattys SL, Jusczyk PW, Luce PA, Morgan JL (1999) Phonotactic and prosodic effects on word segmentation in infants. Cognitive Psychology 38: 465–494. 10334878 10.1006/cogp.1999.0721

[17] Aslin R, Woodward J, LaMendola N, Bever T (1996) Models of word segmentation in fluent maternal speech to infants. In J. Morgan & K. Demuth (Eds.), Signal to syntax: Bootstrapping from speech to grammar in early acquisition. Mahwah, NJ: Lawrence Erlbaum Associates. pp. 117–134

[18] van de Weijer J (1998) Language input for word discovery. Unpublished doctoral dissertation, Max Planck Series in Psycholinguistics 9.

[19] Thiessen E, Saffran J (2003) When cues collide: Use of stress and statistical cues to word boundaries by 7- to 9-month-old infants. Developmental Psychology 39: 706–716. 12859124 10.1037/0012-1649.39.4.706

[20] Lew-Williams C, Saffran JR (2012) All words are not created equal: Expectations about word length guide infant statistical learning. Cognition 122: 241–246. 22088408 10.1016/j.cognition.2011.10.007 PMC3246061

[21] Pelucchi B, Hay JF, Saffran JR (2009) Statistical learning in a natural language by 8-month-old infants. Child Development 80: 674–685. 19489896 10.1111/j.1467-8624.2009.01290.x PMC3883431

[22] Saffran JR, Aslin RN, Newport EL (1996) Statistical learning by eight-month old infants. Science 274: 1926–1928. 8943209 10.1126/science.274.5294.1926

[23] Swingley D (2005) Statistical clustering and the contents of the infant vocabulary. Cognitive Psychology 50: 86–132. 15556130 10.1016/j.cogpsych.2004.06.001

[24] Thiessen ED, Erickson LC (2013) Discovering words in fluent speech: The contribution of two kinds of statistical information. Frontiers in Psychology 3: 590 doi: 10.3389/fpsyg.2012.00590 23335903 10.3389/fpsyg.2012.00590 PMC3547220

[25] Sahni SD, Seidenberg MS, Saffran JR (2010) Connecting cues: Overlapping regularities support cue discovery in infancy. Child Development 81: 727–736. 20573101 10.1111/j.1467-8624.2010.01430.x PMC2892808

[26] Thiessen ED, Saffran JR (2007) Learning to learn: Infants’ acquisition of stress-based segmentation strategies for word segmentation. Language, Learning and Development 3: 73–100.

[27] Johnson EK, Tyler MD (2010) Testing the limits of statistical learning for word segmentation. Developmental Science 13: 339–345. 20136930 10.1111/j.1467-7687.2009.00886.x PMC2819668

[28] Shukla M, White KS, Aslin RN (2011) Prosody guides the rapid mapping of auditory word forms onto visual objects in 6-month-old infants. Proceedings of the National Academy of Sciences 108: 6038–6043. 10.1073/pnas.1017617108 PMC3076873 21444800

[29] Yang CD (2004) Universal grammar, statistics, or both? Trends in Cognitive Sciences 8: : 451– 456. 10.1016/j.tics.2004.08.006 15450509

[30] Johnson EK (2012) Bootstrapping language: Are infant statisticians up to the job? In P. Rebuschat & J. Williams (Eds.). Statistical learning and language acquisition. Mouton de Gruyter. pp. 55–89.

[31] Goldsmith J (2011) The syllable. In J. Goldsmith, J. Riggle, & A. Yu (Eds.). The handbook of phonological theory. Wiley-Blackwell, Oxford, UK. pp. 164–196.

[32] Höhle B, Bijeliac-Babic R, Herold B, Weissneborn J, Nazzi T (2009) Language-specific prosodic preferences during the first half year of life: Evidence from German and French infants. Infant Behavior and Development 3: 262–274. 10.1016/j.infbeh.2009.03.004 19427039

[33] Daland R, Pierrehumbert JB (2011) Learnability of diphone based segmentation. Cognitive Science 35: 119–155. 21428994 10.1111/j.1551-6709.2010.01160.x

[34] Johnson EK, Seidl AH (2008) Clause segmentation by 6-month-olds: A crosslinguistic perspective. Infancy 13: 440–455.

[35] Seidl A, Johnson EK (2008) Boundary alignment enables 11-month-olds to segment vowel initial words from speech. Journal of Child Language 35: 1–24. 18300427 10.1017/s0305000907008215

[36] Shukla M, Nespor M, Mehler J (2006) An interaction between prosody and statistics in the segmentation of fluent speech. Cognitive Psychology 54: 1–32. 16782083 10.1016/j.cogpsych.2006.04.002

[37] Selkirk E (1984) Phonology and syntax: The relation between sound and structure. Cambridge, MA: MIT Press.

[38] Christophe A, Dupoux E, Bertoncini J, Mehler J (1994) Do infants perceive word boundaries? An empirical study of the bootstrapping of lexical acquisition. Journal of the Acoustical Society of America 95: 1570–1580. 8176060 10.1121/1.408544

[39] Christophe A, Mehler J, Sebastián-Gallés N (2001) Perception of prosodic boundary correlates by newborn infants. Infancy 2: 385–394. 10.1207/S15327078IN0203_6 33451208

[40] Hirsh-Pasek K, Kemler-Nelson D, Jusczyk P, Cassidy K, Druss B, et al (1987) Clauses are perceptual units for young infants. Cognition 26: 269–286. 3677573 10.1016/s0010-0277(87)80002-1

[41] Soderstrom M, Seidl A, Kemler Nelson D, Jusczyk PW (2003) The prosodic bootstrapping of phrases: Evidence from prelingual infants. Journal of Memory and Language 49: 249–267.

[42] Gout A, Christophe A, Morgan JL (2004) Phonological phrase boundaries constrain lexical access II. Infant data. Journal of Memory and Language 51: 548–567.

[43] Johnson EK (2003) Word segmentation during infancy: The role of subphonemic cues to word boundaries. Unpublished doctoral dissertation, The Johns Hopkins University.

[44] Johnson EK (2008) Infants use prosodically conditioned acoustic-phonetic cues to extract words from speech. Journal of the Acoustical Society of America 123: EL144–EL148. 18537301 10.1121/1.2908407

[45] Christophe A, Peperkamp S, Pallier C, Block E, Mehler J (2004) Phonological phrase boundaries constrain lexical access: I. Adult data. Journal of Memory and Language 51: 523–547.

[46] Bortfeld H, Morgan JL, Golinkoff RM, Rathbun K (2005) Mommy and me: Familiar names help launch babies into speech-stream segmentation. Psychological Science 16: 298–304. 15828977 10.1111/j.0956-7976.2005.01531.x PMC2981583

[47] Swingley D (2009) Contributions of infant word learning to language development. Philosophical Transactions of the Royal Society B 364: 3617–3632. 10.1098/rstb.2009.0107 PMC2828984 19933136

[48] Endress AD, Mehler J (2010) Perceptual constraints in phonotactic learning. Journal of Experimental Psychology: Human Perception and Performance 36: 235–250. 20121307 10.1037/a0017164

[49] Bergelson E, Swingley D (2012) At 6-9 months, human infants know the meanings of many common nouns. PNAS 109: 3253–3258. 22331874 10.1073/pnas.1113380109 PMC3295309

[50] Tincoff R, Jusczyk PW (1999) Some beginnings of word comprehension in 6-month-olds. Psychological Science 10: 172–175.

[51] Tincoff R, Jusczyk PW (2012) Six month olds comprehend words that refer to parts of the body. Infancy 17: 432–444. 10.1111/j.1532-7078.2011.00084.x 32693484

[52] Johnson EK, Seidl A (2009) At 11 months, prosody still outranks statistics. Developmental Science 12: 131–141. 19120421 10.1111/j.1467-7687.2008.00740.x

[53] Thiessen E, Hill EA, Saffran J (2005) Infant-directed speech facilitates word segmentation. Infancy 7: 53–71. 10.1207/s15327078in0701_5 33430544

[54] Johnson EK (2005) English-learning infants' representations of word-forms with iambic stress. Infancy 7: 95–105. 10.1207/s15327078in0701_8 33430538

[55] Mattys SL, Jusczyk PW (2001) Do infants segment words or recurring contiguous patterns? Journal of Experimental Psychology: Human Perception and Performance 27: 644–655. 11424651 10.1037//0096-1523.27.3.644

[56] Johnson EK, Jusczyk PW, Cutler A, Norris D (2003) Lexical viability constraints on speech segmentation by infants without a lexicon. Cognitive Psychology 46: 65–97. 12646156 10.1016/s0010-0285(02)00507-8

[57] van Heugten M, Johnson EK (2012) Infants exposed to fluent natural speech succeed at cross-gender word recognition. Journal of Speech, Language, and Hearing Research 55: 554–560. 10.1044/1092-4388(2011/10-0347) 22207697

[58] Houston DM, Jusczyk PW (2000) The role of talker-specific information in word segmentation by infants. Journal of Experimental Psychology: Human Perception and Performance 26: 1570–1582. 11039485 10.1037//0096-1523.26.5.1570

[59] Schmale R, Cristià A, Seidl A, Johnson EK (2010) Developmental changes in infants’ ability to cope with dialect variation in word recognition, Infancy. 15: 650–662. 10.1111/j.1532-7078.2010.00032.x 32693460

[60] Hunter MA, Ames EW (1988) A multifactor model of infant preferences for novel and familiar stimuli. In C. Rovee-Collier & L.P. Lipsitt (Eds.), Advances in Infancy Research Volume 5. Norwood, NJ: Ablex, pp. 69–95.

[61] Kidd C, Piantadosi ST, Aslin RN (2012) The Goldilocks Effect: Human infants allocate attention to visual sequences that are neither too simple nor too complex. PLoS One 7(5): e36399 doi:10.1371/journal.pone.0036399 22649492 10.1371/journal.pone.0036399 PMC3359326

[62] Fernald A, Mazzie C (1991) Prosody and focus in speech to infants and adults. Developmental Psychology 27: 209–221.

[63] Phillips J (1973) Syntax and vocabulary of mothers’ speech to young children: Age and sex comparisons. Child Development 44: 182–185.

[64] Brent MR, Siskind JM (2001) The role of exposure to isolated words in early vocabulary development. Cognition 8: B33–44. 10.1016/s0010-0277(01)00122-6 11376642

[65] Shi R, Morgan J, Allopenna P (1998) Phonological and acoustic bases for earliest grammatical category assignment: A cross-linguistic perspective. Journal of Child Language 25: 169–201. 9604573 10.1017/s0305000997003395

[66] Nazzi T, Dilley LC, Jusczyk AM, Shattuck-Hufnagel S, Jusczyk PW (2005) English-learning infants' segmentation of verbs from fluent speech. Language and Speech 48: 279–298. 16416938 10.1177/00238309050480030201

[67] Kuhl PK (1979) Speech perception in early infancy: Perceptual constancy for perceptually dissimilar vowel categories. Journal of the Acoustical Society of America 66: 1168–1679. 10.1121/1.383639 521551

[68] Singh L, Nestor SS, Bortfeld H (2008) Overcoming effects of variation on infant word recognition: influences on word familiarity. Infancy 13: 57–74. 21088702 10.1080/15250000701779386 PMC2982147

[69] Shi R, Cutler A, Werker JF, Cruickshank M (2006) Frequency and form as determinants of functor sensitivity in English acquiring infants. JASA 119: EL61–EL67. 10.1121/1.2198947 16838552

[70] Johnson EK, Sohail J, Zamuner T (2012) How transitional probabilities and the edge effect contribute to listener’s phonological bootstrapping success. Talk presented at the 2012 International Conference on Infant Studies, Minneapolis, Minnesota.

