# Supplementary material for: The Edge Factor in Early Word Segmentation: Utterance-Level Prosody Enables Word Form Extraction by 6-Month-Olds
Source: PLoS One. 2014 Jan 8;9(1):e83546. doi: 10.1371/journal.pone.0083546 (PMC3885442; doi:10.1371/journal.pone.0083546)
Supplement: List S1 — Sample familiarization passages with alternating utterance-initial and utterance-final target words (Experiment 1, 3, and 4). (DOC) [file pone.0083546.s001.doc]

# List S1

Geff runs the big circus in Toronto.

At the silly circus I like low Geff.

Geff wants to juggle out there all day long.

It is such bad luck to have a rough Geff

Geff has a great time making sugar snaps.

I’ll have to tell her that we know Geff.

Sample passages with utterance-medial target words

(Experiment 1, Medial Condition)

I like how Geff runs the circus.

I wonder if Geff wants to juggle too.

We know Geff has a great time.

We’re sure Geff would love to learn.

I think Geff could do a great job.

They say Geff hires clowns all year.

Sample passages with utterance-initial target words

(Experiment 2, Initial Condition)

Geff runs the big circus in Toronto.

Geff wants to juggle out there all day long.

Geff has a great time making sugar snaps.

Geff would love to learn to bake lasagna.

Geff could do a great job fixing our school.

Geff hires clowns to drive a tiny car around.

Sample passages with utterance-final target words

(Experiment 2, Final Condition)

At the circus I like low Geff.

It is such bad luck to have a rough Geff.

We’ll have to tell her that we know Geff.

The lion tamer knows how to ensure Geff.

The elephants always want to thank Geff.

The clowns start dancing when they say Geff.
